# CCN4 (WISP-1) reduces apoptosis and atherosclerotic plaque burden in an ApoE mouse model

**DOI:** 10.1016/j.atherosclerosis.2024.118570

**Published:** 2024-08-26

**Authors:** Helen Williams, Steven Simmonds, Andrew Bond, Alexandros Somos, Ze Li, Tessa Forbes, Rosaria Bianco, Celyn Dugdale, Zoe Brown, Helen Rice, Andrew Herman, Jason Johnson, Sarah George

**Affiliations:** a Bristol Heart Institute, Bristol Medical School, University of Bristol, UK; b KU Leuven, Belgium; c Flow Cytometry Facility, School of Cellular & Molecular Medicine, University of Bristol, UK

**Keywords:** CCN-4/ WISP-1, Atherosclerosis, Wnt pathway, Apoptosis, Macrophages, VSMCs, Apolipoprotein E

## Abstract

**Background and aims:**

CCN4/WISP-1 regulates various cell behaviours that contribute to atherosclerosis progression, including cell adhesion, migration, proliferation and survival. We therefore hypothesised that CCN4 regulates the development and progression of atherosclerotic plaques.

**Methods:**

We used a high fat fed *ApoE*^−/−^ mouse model to study atherosclerotic plaque progression in the brachiocephalic artery and aortic root. In protocol 1, male *ApoE*^−/−^ mice with established plaques were given a CCN4 helper-dependent adenovirus to see the effect of treatment with CCN4, while in protocol 2 male CCN4^−/−^ *ApoE*^−/−^ were compared to CCN4^+/+^*ApoE*^−/−^ mice to assess the effect of CCN4 deletion on plaque progression.

**Results:**

CCN4 overexpression resulted in reduced occlusion of the brachiocephalic artery with less apoptosis, fewer macrophages, and attenuated lipid core size. The amount of plaque found on the aortic root was also reduced. CCN4 deficiency resulted in increased apoptosis and occlusion of the brachiocephalic artery as well as increased plaque in the aortic root. Additionally, *in vitro* cells from CCN4^−/−^ *ApoE*^−/−^ mice had higher apoptotic levels. CCN4 deficiency did not significantly affect blood cholesterol levels or circulating myeloid cell populations.

**Conclusions:**

We conclude that in an atherosclerosis model the most important action of CCN4 is the effect on cell apoptosis. CCN4 provides pro-survival signals and leads to reduced cell death, lower macrophage number, smaller lipid core size and reduced atherosclerotic plaque burden. As such, the pro-survival effect of CCN4 is worthy of further investigation, in a bid to find a therapeutic for atherosclerosis.

## 1 Introduction

CCN4 (WISP-1: Wnt1 inducible signalling pathway protein 1) is a member of the CCN family of secreted signalling proteins [1]. The CCN family modulates various cell behaviours including adhesion, migration, proliferation, differentiation and survival [2,3]. They also have roles in the pathological processes of injury repair, fibrosis, inflammation and cancer [4]. Previous work by our group and others has shown that CCN4 plays a role in many of the processes that may be relevant to the progression and composition of atherosclerotic plaques, such as cell migration, proliferation and apoptosis [3,5–7].

Initially CCN4 was studied in the context of various cancer models. Although CCN4 has been widely shown to be pro-proliferative in a number of different cancer models, including prostate, eosophageal and oral cancers, conflicting data has been noted in other cancers (as summarised by Gurbuz & Chiquet-Ehrismann [8] and Nivison and Meier [9]). For example, in breast and ovarian cancer models, some studies showed a beneficial effect of CCN4 in reducing cancers, while others demonstrated the opposite [8,9]. The role of CCN4 in cancer appears to be dependent on both the cell type and model utilised, and consequently further research is required to clarify its role in these models. The ability of CCN4 to act as an autocrine and paracrine factor and act via both the canonical Wnt/β-catenin pathway and through various integrins, NFκB and MMP2, depending on the cell type studied [10], may enable CCN4 to cause diverse effects.

More recently, the role of CCN4 has been studied in the cardiovascular system and in cells relevant to the study of atherosclerosis, including vascular smooth muscle cells (VSMCs) and macrophages. Liu et al. showed that treatment of rat VSMCs with recombinant CCN4 induced cell migration and proliferation and that the effect on migration was mediated through integrin α5β1 [3]. Levels of CCN4 were associated with levels of both elastin and osteopontin, indicative that CCN4 acts to switch the VSMCs to a more contractile phenotype. We have previously shown similar effects of CCN4 on migration in mouse VSMCs [7]. Moreover, CCN4 was induced by Wnt2, both *in vivo* and *in vitro*, via a β-catenin dependent pathway and the Frizzled-6 receptor [7]. CCN4 led to an integrin dependent increase in cell migration *in vitro* and a significant increase in cell migration *in vivo* in the mouse carotid artery ligation model [7].

CCN4 acts as a pro-survival factor suppressing apoptosis in various cell types including VSMCs [6,11]. Since VSMC apoptosis within the atherosclerotic plaque leads to vulnerability to plaque rupture, apoptosis of VSMCs is considered a driving factor in atherosclerotic plaque progression and rupture. We previously showed that CCN4 was increased in human unstable atherosclerotic plaques compared to stable plaques [6]. Additionally, we observed that the CREB-dependent upregulation of CCN4 was lost in mice with age, therefore reducing the pro-survival effect. This could potentially lead to formation of more vulnerable atherosclerotic plaques in an ageing population [5]. March- and et al. [12] and our own group [6,7] confirmed that CCN4 is present in both human arterial cells and human atherosclerotic plaques respectively, showing that this pathway is present in human tissues as well as mouse.

The role of CCN4 in monocytes and macrophages has been less well studied, but evidence is emerging that a role is present for CCN4 inducing monocyte recruitment [13] and increased inflammatory response in macrophages as a response to injury and to promote wound repair. CCN4 induced monocyte adhesion appears to be mediated by a variety of integrins, including αVβ5, αVβ3 and β1 integrins [3,10, 14–16].

These multiple possible roles for CCN4 in the different processes associated with atherosclerosis make it an intriguing candidate for further investigation. In this study we utilised the high fat fed Apolipoprotein E knockout mouse (*ApoE*^−/−^) model of atherosclerosis. We used a helper-dependent adenovirus encoding CCN4 to enhance circulating levels, and CCN4-deficient mice, to monitor the effects of both increasing and knocking out CCN4 on the progression of atherosclerosis. We have also studied the effect of increasing or deleting CCN4 on macrophage behaviour *in vitro* to assist with determining the underlying mechanism of action of CCN4 in the regulation of atherosclerosis.

## 2 Materials and methods

The raw data that support the findings of this study are available from the corresponding author upon reasonable request.

### 2.1 Husbandry

The housing and care of all the animals and the procedures used in this study were performed in accordance with the guidelines and regulations of the University of Bristol and the United Kingdom Home Office. The investigation conforms to the Guide for the Care and Use of Laboratory Animals published by the US National Institutes of Health (NIH Publication No. 85-23, revised 1996). The study was carried out using the NC3Rs ARRIVE guidelines and was approved by the animal welfare and ethical review body (AWERB). Mice were caged in groups of 2–5 with environmental enrichment of houses, tubes and chewing blocks.

### 2.2 Transgenic mice

CCN4 homozygous deficient mice (*CCN4*^−/−^) mice on a C57/bl6J background from more than 10 backcrosses were a kind gift from Marian Young (NIH, Bethesda, MD). Apolipoprotein E^−/−^ mice were purchased from Charles River UK (criver.com). Mice were bred at the University of Bristol breeding facility under specific pathogen free conditions to produce *CCN4*^−/−^ *ApoE*^−/−^ double knockout mice and *CCN4*^+/+^*ApoE*^−/−^ wild-type littermate controls. Power analysis was used to indicate that 15 mice were required per group to show a significant difference in plaque size. Animals were excluded if they did not survive to experiment end point. Cages of animals were randomised to groups using a random sequence generator (random.org).

### 2.3 In vivo experiments

Two protocols were performed to elucidate the effect of CCN4 deletion and CCN4 overexpression. These protocols differed in their time-course and study approach therefore it is important to note that the controls are not comparable between the two studies.

Protocol 1: Treatment of established atherosclerosis by over-expression with CCN4.

Helper dependent adenoviruses for CCN4 and a scrambled control were designed and purchased from Kazuhiro Oka at Gene Vector Core, Baylor College of Medicine, USA. Male *ApoE*^−/−^ mice were fed a high fat diet (custom high fat diet consisting of 22 % fat from lard and 0.15 % cholesterol purchased from Testdiet.com product code T-5W83-1816018-270, full composition shown in Supplementary Table 1) from 7 to 8 weeks of age for 10 weeks to induce atherosclerosis in the mice prior to treatment. We have shown previously that this produces atheroscle-rotic plaques with characteristics similar to those seen in human coronary arteries [17]. They were then infected with either HD-Ad CCN4 or control virus before being fed high fat diet for a further 6 weeks. Mice were subjected to a tail vein injection of 1 × 10^11^ pfu of HD-Ad CCN4 or control virus while under isofluorane anaesthesia (3 % isofluorane in O_2_).

Protocol 2: The effect of CCN4 deletion on atherosclerosis development.

Male *CCN4*^−/−^ *ApoE*^−/−^ mice and *CCN4*^+/+^*ApoE*^−/−^ mice were fed a high fat diet from 7 to 8 weeks of age for 10 weeks duration.

At the end of the protocols all mice were culled using an IP overdose of sodium pentobarbital (500 mg/kg) followed by exsanguination by perfusion fixation with PBS followed by 10 % formalin in PBS. Outflow of fixative was via transected jugular veins. Vessels and tissues were fixed in 10 % formalin in PBS overnight before processing and paraffin wax embedding for histology. The experimenter was aware of which mice received which treatment, but all analysis was performed while blinded to treatment group.

### 2.4 Cholesterol assay

Total cholesterol, HDL cholesterol and vLDL/LDL cholesterol were measured from mouse plasma samples using the kit and protocol provided by Abcam (ab65390).

### 2.5 CCN4 ELISA

Plasma samples were diluted 1:2 in PBS and subjected to an ELISA for human CCN4 protein (R&D systems, DY1627), all reagents were supplied by the manufacturer, except for use of our own substrate Sigma fast o-phenylenediamine dihydrochloride (OPD).

### 2.6 Histochemistry, immunohistochemistry and immunofluorescence

All analysis was performed while blinded to treatment group. We chose to examine atherosclerotic plaque burden in the braciocephalic artery, as well as the aortic root valve leaflets, as many studies from our group and others have shown these to be well-defined sites with consistent levels of disease, due to the flow characteristics experienced here [17,18]. 3 μm sections of mouse brachiocephalic artery were cut onto Superfrost slides for histological stains and onto Superfrost Plus slides for immunohistochemistry and immunofluorescence. In all brachiocephalic artery samples sections were analysed at the same location, immediately after the bifurcation from the aortic arch. This site is selected as we have previously shown that large plaques are consistently located here presumably due to the turbulent flow adjacent to the bifurcation. In all aortic root samples, sections were analysed at the location at which all valve leaflets were visible in the section. EVG stained sections were used for visualisation and measurement of the vessel and plaque by image analysis (using Image Pro or ImageJ). Picrosirius red staining was used to visualise collagen. Immunohisto-chemistry was performed to visualise α-smooth muscle actin (Sigma A2547, 3.1 μg/ml), macrophages (GSL Griffonia simplicifolia lectin Vector Labs B1205, 2.5 μg/ml endothelial cells (CD31, Dako, clone JC70A), apoptosis (cleaved caspase 3, R&D Systems, AF835, 1 μg/ml) and proliferation (PCNA, proliferating cell nuclear antigen, Abcam 18197, 1 μg/ml). Dual staining was performed to assess the co-location of apoptosis with macrophages. Sections of liver were stained with haematoxylin and eosin (H&E) to assess general liver morphology and BiP (binding immunoglobulin protein) to quantify whether oxidative stress was affected as a result of CCN augmentation. Non-immune IgG of the same species as the primary was used as negative control in all protocols at the same concentration as the primary antibody to demonstrate the specificity of the protocol.

### 2.7 Flow cytometry

The Cytek® Tonbo™ Mouse Myeloid identification kit (Tube 2 - SKU 95-K001-T025) was utilised for phenotyping. The samples and single stained reference controls were acquired on a 4-laser Cytek Aurora Full spectrum analyser (V-B-YG-R), running SpectroFlo (V3.2.1). Once acquired, recorded samples were unmixed (compensated) within SpectroFlo and exported to FlowJo (V10.10.0) for analysis.

Sample Preparation – Terminal blood samples were taken from *ApoE*^−/−^ *CCN4*^+/+^ and *ApoE*^−/−^ *CCN4*^−/−^ mice using heparin coated syringes. 100 μl blood was frozen in Cell Banker 2 (Zenogen Pharma 11914). Frozen samples were thawed rapidly, transferred to 5 ml FACS tubes (Falcon - 06321012) and washed with 1 ml PBS before being centrifuged at 1500 rpm at 4 °C for 5 min. The supernatant was discarded and 2 ml of ACK lysis buffer (Gibco – A10492-01) was added and the tubes agitated for 3 min to lyse red blood cells (RBCs). The samples were then washed with 3 ml PBS before being spun down again at 400g at 4 °C for 5 min and the supernatant removed. Antibody cocktail was created using 5 μl per test Cytek dye. A live/dead dye (Ghost Dye Violet 540) cocktail was also created using a dilution factor of 1:250 from the stock with PBS. These were wrapped in foil and kept on ice during the entire staining process.

Samples were stained with 5 μl of the live/dead cocktail, vortexed lightly and incubated for 20 min at 4 °C in the dark. After incubation, the samples were washed with 3 ml staining buffer (Biolegend - 420201) and centrifuged at 400g for 5 min at 4 °C before the supernatant was decanted. Samples were then treated with 0.5 μl TruStain Monocyte Blocker (Biolegend – 426102) and 5 μl TruStain FcX™ PLUS (Biolegend - 156603) and incubated for 10 min in the dark at 4 °C before 55 μl of the antibody cocktail was added to each sample, vortexing lightly after the addition of cocktail. After the incubation for 30 min in the dark at 4 °C, the samples were washed again with 3 ml staining buffer and centrifuged at 400g for 5 min at 4 °C, and resuspended in staining buffer to create a final sample volume of 200 μl.

Single Stain Reference Controls – For the single stain reference controls we used Ly5 mouse splenocytes resuspended in PBS in a concentration of ∼10million cells/ml. Each reference control used ∼500k cells (50 μl). For the live/dead control, half the cells were heat killed before staining to ensure a distinct positive and negative population were present. All the single colour controls were washed with 3 ml PBS and centrifuged at 400g for 5 min at 4 °C. The supernatant was discarded before each control was treated with Fc block (except for Live/Dead) and left to incubate in the dark for 10 min at 4 °C. Each control was stained with 3 μl of one of the Cytek dyes including Live/Dead, incubated for 20 (Live/dead) or 30 (all other markers) minutes at 4 °C in the dark, before being washed with 3 ml staining buffer and centrifuged at 400g for 5 min at 4 °C and the supernatant discarded. Each single stain was then resuspended with staining buffer to make a final volume of 150 μl.

### 2.8 In vitro study of macrophages

Monocytes were obtained from human blood or from mouse bone marrow from *CCN4*^+/+^ and *CCN4* ^−/−^ mice.

### 2.9 Culture of human monocytes

Peripheral blood was collected from healthy human volunteers in accordance with the Regional Ethics Committee (NRES#10/H0107/32). Blood was diluted with an equal volume of sterile PBS, stratified on Ficoll-PaqueTM Plus. The peripheral blood monocytes were collected, washed and seeded in 20 ng/ml M-CSF in 10 % FCS/RPMI 1640. The resultant adherent monocyte cells were left to mature for 7 days before use.

### 2.10 Culture of mouse monocytes

Mice were euthanized using a schedule 1 method and femur and tibia removed by dissection. A 26-gauge needle was inserted into the bone marrow cavity of each bone and the cavity flushed with 2 ml HBSS media. Cells were washed, seeded and matured as per human cells.

### 2.11 Treatment with oxidised LDL (ox-LDL)

Macrophages were treated concurrently with 20 μg/ml ox-LDL and 500 ng/ml CCN4 for 48 h to investigate LDL uptake, foam cell formation and effect on apoptosis.

### 2.12 Visualisation of intracellular lipids with Oil red O

Oil Red O Staining was performed using 2 % Oil Red O in isopropanol (Sigma O0625) followed by nuclear stain with Mayer’s haematoxylin (Merck, HX093785) and mounted using PVP aqueous mountant.

### 2.13 Immunocytochemistry for cleaved caspase 3

Apoptosis was measured using cleaved caspase-3 (CC3) immunocytochemistry. Cells were cytospun onto slides and stained using CC3 antibody at 1 μg/ml (R&D Systems AF835) [19].

### 2.14 Statistical analysis

Results are expressed as mean ± SEM. Data was first analysed for normality before selection of the appropriate statistical test in each case, students t-test for normally distributed data and Mann-Whitney test for non-normally distributed. Paired or unpaired analysis was used as appropriate and Kruskal-Wallis test with Dunn’s multiple comparison test was used for multiple comparisons. A significant difference was accepted when *p<*0.05.

## 3 Results

**Fig. 1:**
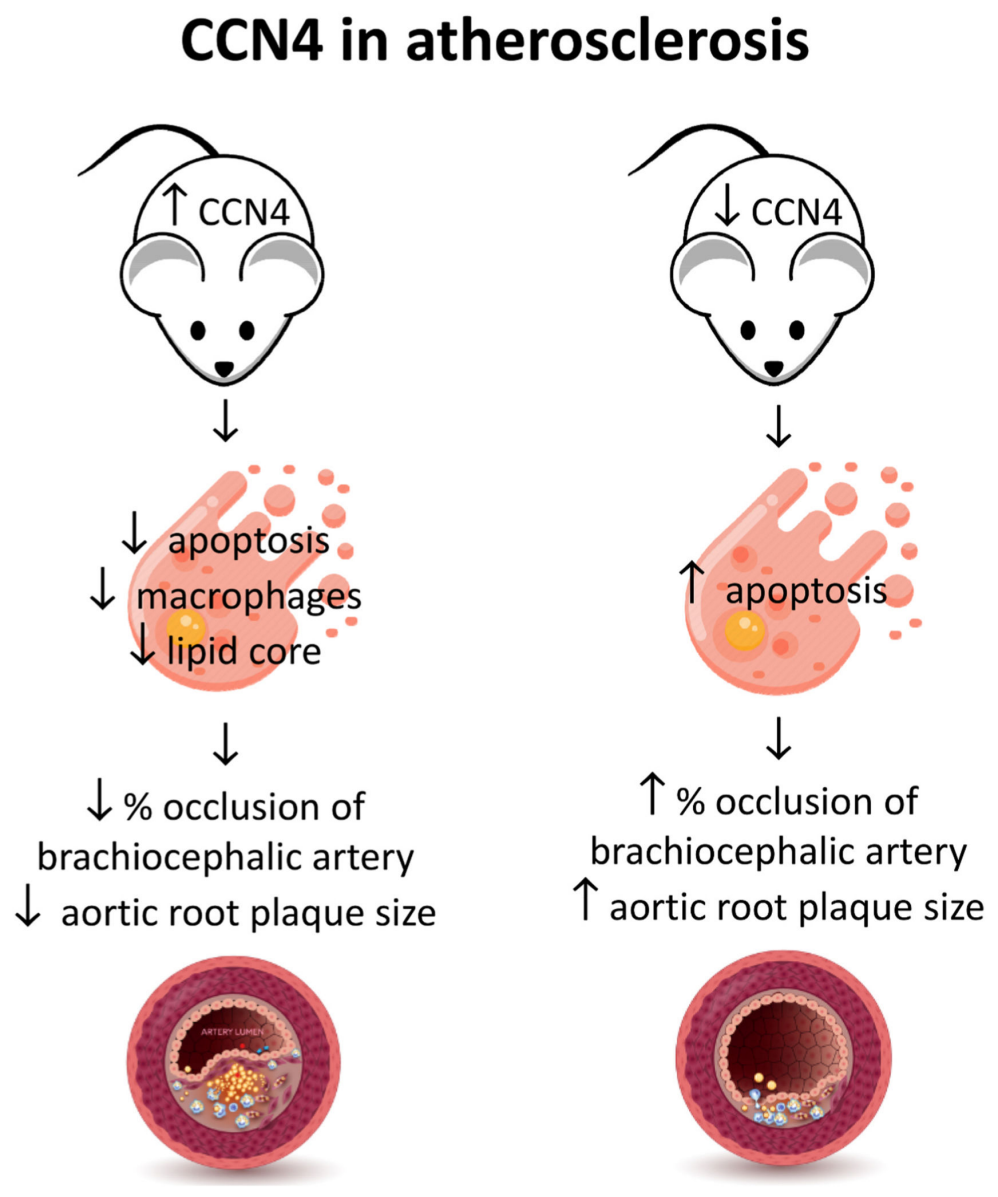
Graphic abstract summarising the findings of the paper.

A graphical summary of our results is shown in Fig. 1.

### 3.1 CCN4 overexpression virus reduced brachiocephalic artery occlusion, lipid core size, apoptotic rate, number of macrophages in the brachiocephalic artery and atherosclerotic lesion size in the aortic root

**Fig. 2:**
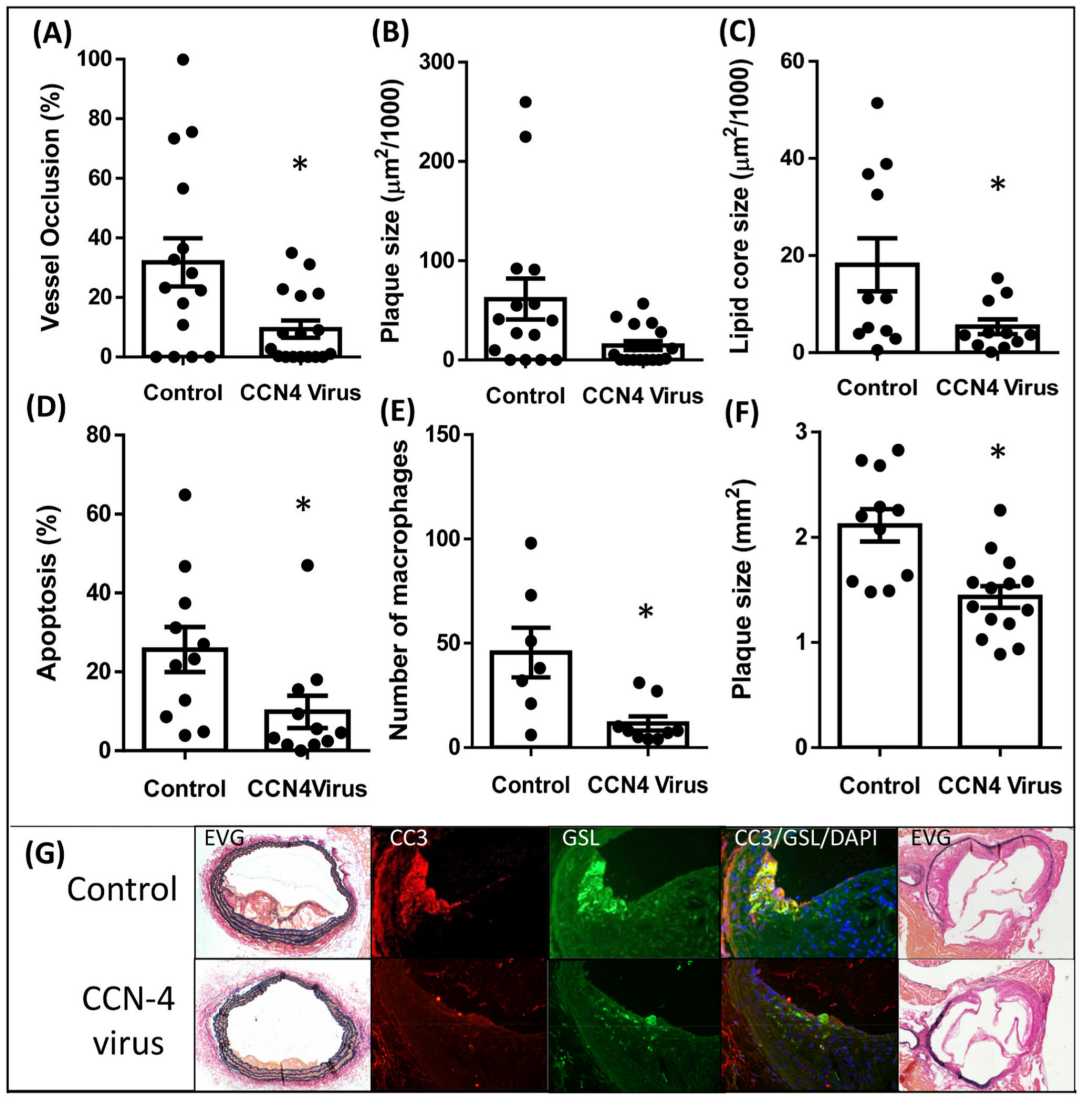
CCN4 overexpression decreased % vessel occlusion by atherosclerotic plaque, lipid core size, apoptosis and macrophage number in mouse brachiocephalic arteries and decreased atherosclerotic lesion size in aortic roots. Brachiocephalic arteries and aortic roots from ApoE-deficient (*ApoE*^−/−^) mice were treated with either CCN4 overexpression virus or a non-functional control virus. The percentage occlusion of the vessel (A), plaque size (B) and lipid core size (C) in brachiocephalic arteries and the plaque size in aortic root (F) were quantified using image analysis of EVG stained sections. % Apoptosis was measured by cleaved caspase-3 immunohistochemistry CC3 (D). Macrophages were stained using GSL and counted (E). Representative images are shown (G) for CCN4^+/+^ in the top row and *CCN4* ^−/−^ in the bottom row. Sections were stained with EVG to show vessel morphology or IHC was performed for CC3 to show apoptotic cells in red and GSL to show macrophages in green. Nuclei were stained blue with DAPI. Scale bars represent 200 μm in brachiocephalic EVG, 50 μm in CC3/GSL and 200 μm in aortic root EVG. *p<0.05 *vs*. control, *p* = 0.010 n = 15 for vessel occlusion (A), *t*-test, *p* = 0.070 n = 15 for plaque size (B), Mann-Whitney test, *p* = 0.036 n = 11 for lipid core size (C), *t*-test, *p* = 0.015 n = 11 for % apoptosis (D), Mann-Whitney test, *p* = 0.010 n = 7–10 for macrophage numbers (E), Mann-Whitney test, *p* = 0.001 n = 11–14 for aortic root lesion area (F), *t*-test. (For interpretation of the references to colour in this figure legend, the reader is referred to the Web version of this article.)

Following high fat feeding of *ApoE*^−/−^ mice, atherosclerotic plaques developed in the brachiocephalic arteries and aortic roots. Over-expression of CCN4 using a helper dependent adenovirus resulted in a significant increase in plasma levels of CCN4 at both 1 and 6 weeks compared to administration of control virus (Supplementary Fig. 1). Increased levels of plasma CCN4 resulted in a reduction in the percentage occlusion of the brachiocephalic artery compared to control mice (Fig. 2A). These plaques also had significantly smaller lipid cores (Fig. 2C). There was no change in vessel area, cell density, proliferation, cell number, number of smooth muscle cells, endothelial coverage, or collagen content of the plaques (Table 1). Overexpression of CCN4 significantly reduced the proportion of apoptotic cells in HD-Ad CCN4 infected mice (Fig. 2D) and there was also an attenuation in the number of macrophages (Fig. 2E) in the HD-Ad CCN4 infected mice compared to control virus infected mice. Overexpression of CCN4 resulted in decreased aortic root atherosclerotic plaque size (Fig. 2F). Macrophage content was not correlated with plaque size (data not shown). Dual staining of macrophages showed that macrophages were observed in areas of apoptosis with some GSL positive cells also staining positive for CC-3 (Fig. 2G). There was no significant change in total cholesterol, LDL/vLDL or HDL cholesterol between the groups (Table 2). Administration of the CCN4 virus did not adversely affect liver pathology as assessed by H&E staining for general morphology and BiP staining for oxidative stress (Supplementary Fig. 2).

### 3.2 CCN4 deficiency increased brachiocephalic artery occlusion and apoptotic rate in the brachiocephalic artery and atherosclerotic plaque size in the aortic root

**Fig. 3:**
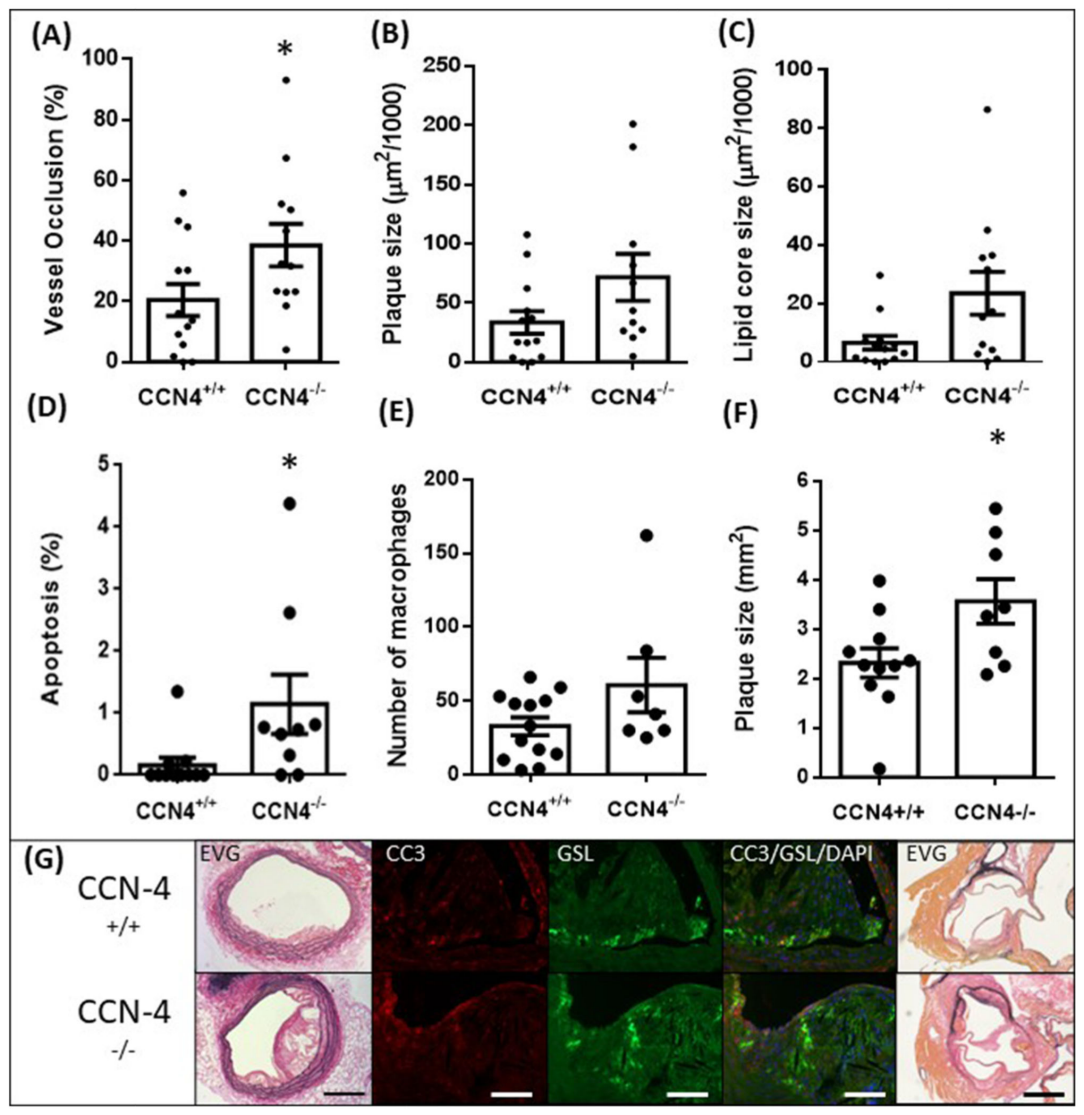
CCN4 deletion increased % vessel occlusion by atherosclerotic plaque and apoptosis in mouse brachiocephalic arteries and increased atherosclerotic lesion size in aortic roots. Brachiocephalic arteries and aortic roots from ApoE-deficient/CCN-4 deficient (*CCN4* ^−/−^) mice were compared to those from ApoE-deficient mice *(CCN-4*^+/+^). The percentage occlusion of the vessel (A), plaque size (B) and lipid core size (C) in brachiocephalic arteries and the plaque size in aortic root (F) were quantified using image analysis of EVG stained sections. % Apoptosis was measured by cleaved caspase-3 immunohistochemistry CC3 (D). Macrophages were stained using GSL and counted (E). Representative images are shown (G) for *CCN4*^+/+^ in the top row and *CCN4* ^−/−^ in the bottom row. Sections were stained with EVG to show vessel morphology or IHC was performed for CC3 to show apoptotic cells in red and GSL to show macrophages in green. Nuclei were stained blue with DAPI. Scale bars represent 200 μm in brachiocephalic EVG, 50 μm in CC3/GSL and 200 μm in aortic root EVG. *p<0.05 *vs*. control (*CCN4*+/+), *p* = 0.049 n = 15 for vessel occlusion (A), *t*-test, *p* = 0.083 n = 15 for plaque size (B), *t*-test, *p* = 0.077 n = 12–13 for lipid core size (C), Mann-Whitney test, *p* = 0.012 n = 9–11 for % apoptosis (D), Mann-Whitney test, *p* = 0.194 n = 7–13 for macrophage numbers (E), Mann-Whitney test, *p* = 0.028 n = 11 and 8 mice for *CCN4*+/+ and *CCN4*^−/−^ respectively for aortic root lesion area (F), *t*-test. (For interpretation of the references to colour in this figure legend, the reader is referred to the Web version of this article.)

*CCN4*^−/−^ mice had significantly greater % occlusion of the vessel compared to *CCN4*^+/+^ littermate controls (Fig. 3A). Deficiency in CCN4 did not significantly alter the vessel area, cell density, proliferation, cell number, number of smooth muscle cells, endothelial coverage, or collagen content of the brachiocephalic artery plaques (Table 2). However, a significant increase in the proportion of apoptotic cells in these plaques was detected compared to controls (Fig. 3D). Deficiency in CCN4 did not significantly alter the number of macrophages (Fig. 3E). The aortic root atherosclerotic plaque size was increased in mice deficient in CCN4 (Fig. 3F). Macrophage content was not correlated with plaque size (data not shown). As with the overexpression study, some of the macrophages were found to be apoptotic following dual staining with GSL and CC-3. There was no significant change in total cholesterol, LDL/vLDL or HDL cholesterol between the groups (Table 2).

### 3.3 CCN4 increased cell survival in macrophages

**Fig. 4:**
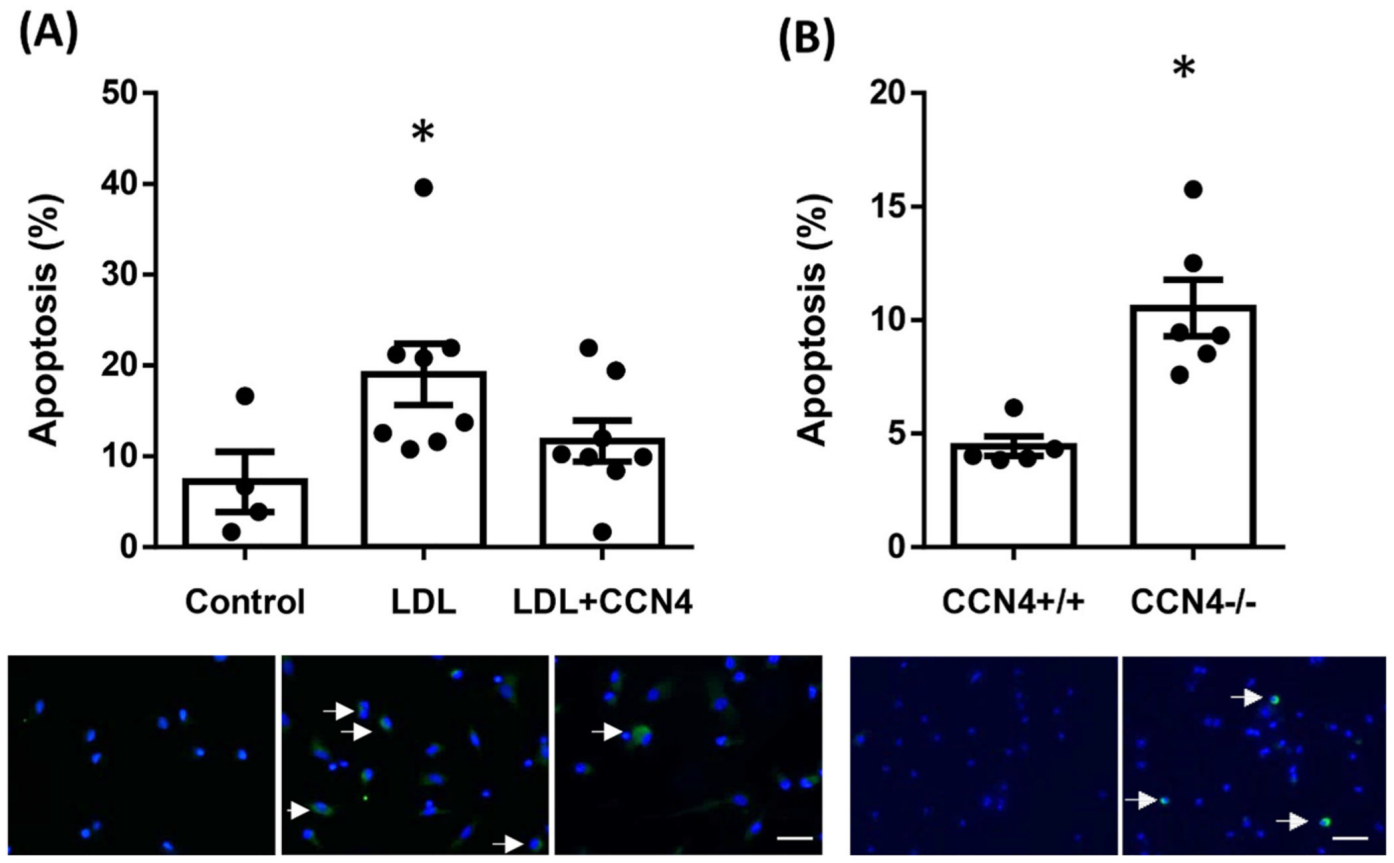
The presence of CCN4 resulted in loss of the LDL dependent increase in apoptosis in human macrophages, while in mouse macrophages *CCN4*^−/−^ macrophages had significantly more apoptosis compared to *CCN4*^+/+^ controls. (A) Primary human macrophages were treated with 20 μg/ml ox-LDL in the presence or absence of 500 ng/ml CCN4. The percentage of CC3 positive cells (apoptotic cells) was quantified after 48 h. LDL treatment led to a significant increase in apoptosis, while concurrent treatment with LDL and CCN4 was not significantly different to control. *p<0.05 *vs*. control, *p* = 0.024 n = 4 and 8, Kruskal-Wallis test with Dunn’s multiple comparison test. (B) Primary murine macrophages from *CCN4*^−/−^ and *CCN4*^+/+^ control mice were treated with 20 μg/ml ox-LDL to induce apoptosis. The percentage of CC3 positive cells was quantified after 48 h *p<0.004 n = 5&6 *vs. CCN4*^+/+^ control mice, Mann-Whitney test. Representative images illustrate CC3 in green and nuclei are stained blue with DAPI. Arrows indicate some positive cells, scale bars represent 50 μm. (For interpretation of the references to colour in this figure legend, the reader is referred to the Web version of this article.)

Human cells cultured with ox-LDL exhibited a significantly enhanced apoptotic rate, but co-incubation with LDL and CCN4 resulted in no such significant increase (Fig. 4A). While mouse macrophages deficient in CCN4 had an increased apoptotic rate compared to wild-type macrophages (Fig. 4B), suggesting that CCN4 is a pro-survival factor in both human and mouse macrophages.

### 3.4 CCN4 did not affect foam cell formation

**Fig. 5:**
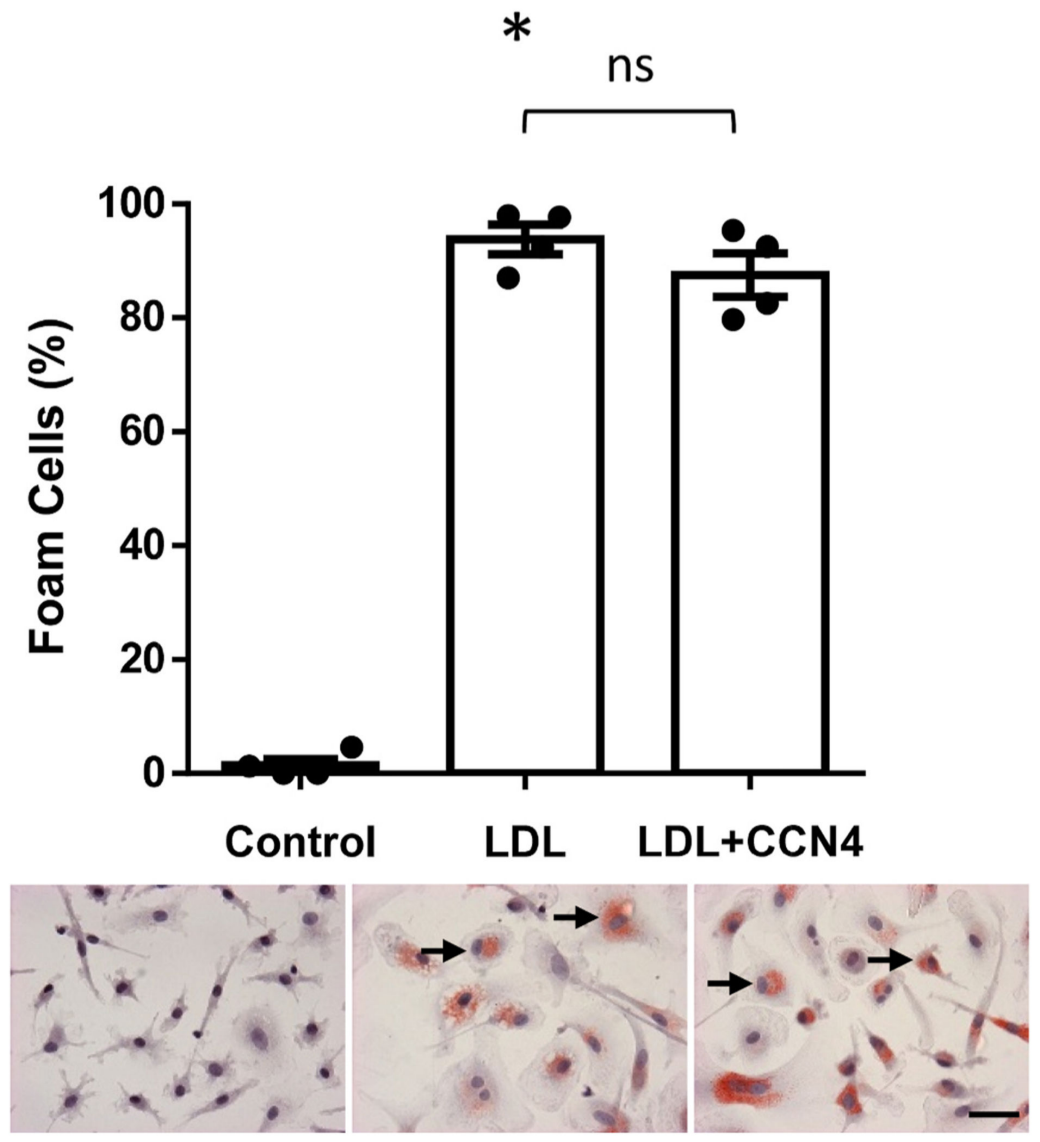
CCN4 did not affect foam cell formation in human macrophages. Primary human macrophages were treated with 20μg//ml ox-LDL in the presence or absence of 500 ng/ml CCN4. The percentage of Oil Red O positive cells was quantified after 48 h *p<0.05 *vs*. control, ns = no significant difference, *p* = 0.005 n = 4, Kruskal-Wallis test with Dunn’s multiple comparison test. Representative images are shown below each graph, positive cells are red and nuclei are stained blue with haematoxylin. Arrows indicate some positive cells, scale bar represents 50 μm. (For interpretation of the references to colour in this figure legend, the reader is referred to the Web version of this article.)

Incubation of human macrophages with CCN4 did not affect their ability to become foam cells in response to incubation with ox-LDL (Fig. 5). Similarly, mouse macrophages from CCN4 deficient mice had a comparable ability to become foam cells to macrophages from control mice (% lipid uptake was 40.25 ± 3.03 % in CCN4 deficient macrophages compared to 42.45 ± 4.79 % in macrophages from wild type mice), suggesting that CCN4 levels do not affect macrophage lipid up-take, so the pro-survival effect observed was not due to changes in lipid uptake.

### 3.5 CCN4 deficiency did not affect % of circulating myeloid cells or inflammatory monocytes

**Fig. 6:**
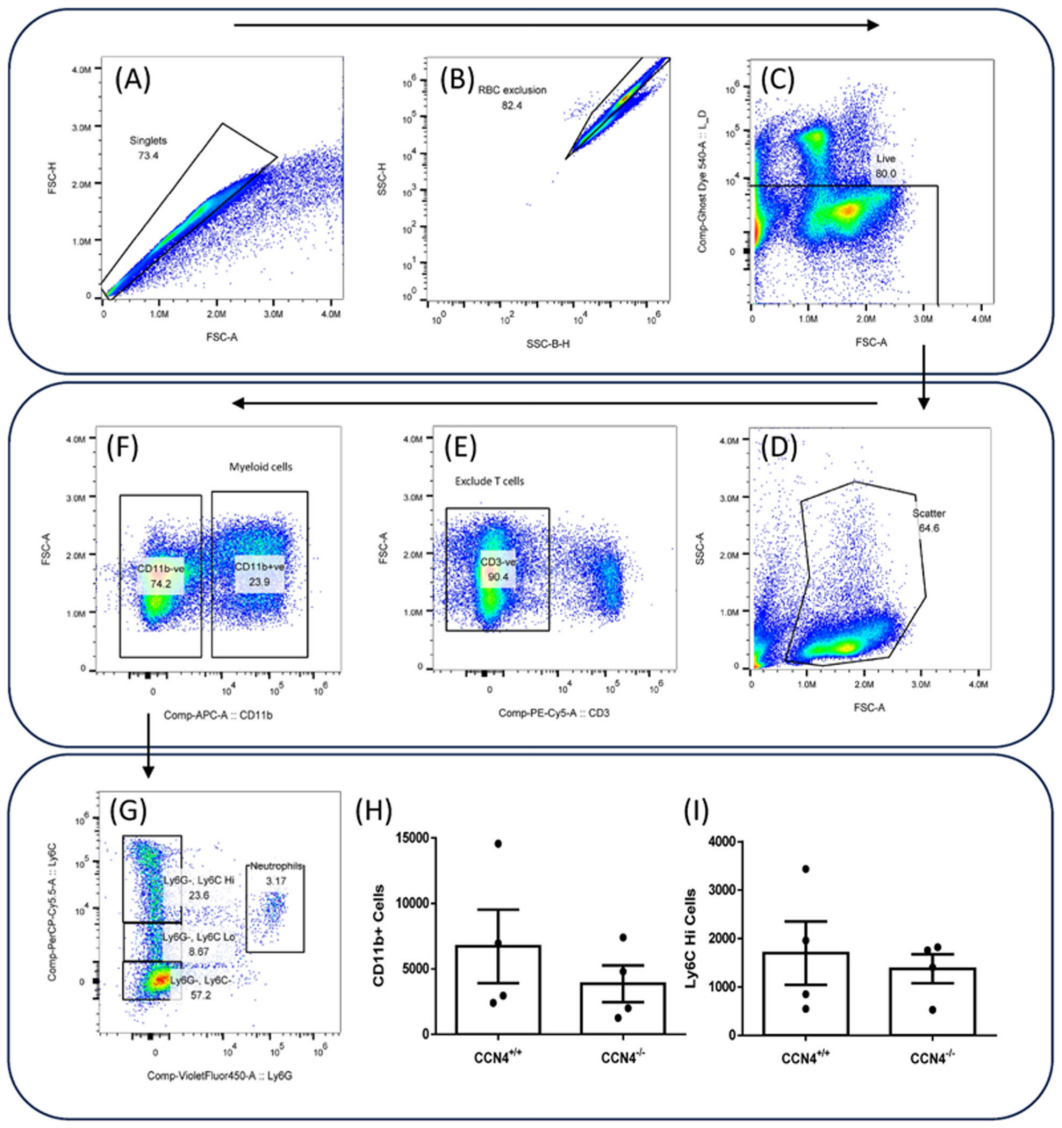
CCN4 knockout did not change the proportion of inflammatory to non-inflammatory circulating monocytes. Flow cytometry was performed using a panel of antibodies to identify leukocyte subtypes. Cells were analysed using the following gating strategy: include only single cells (A), exclude red blood cells (B), include only live cells (C), include appropriate cell size and exclude debris (D), exclude CD3^+^ T cells (E), include CD11b + myeloid cells (F), exclude Ly6G + neutrophils (G) to leave only monocytes. Monocytes were then divided into Ly6C Hi inflammatory monocytes and Ly6C Lo non-inflammatory monocytes (G). CD11b + myeloid cell number (H) and Ly6C + inflammatory monocyte number (I). Mann-Whitney test *p* = 0.49 for CD11b (H) and *p* = 0.66 for Ly6C Hi (I), n = 4. (For interpretation of the references to colour in this figure legend, the reader is referred to the Web version of this article.)

Flow cytometry of terminal blood samples from *ApoE*^−/−^ *CCN4*^−/−^ and *ApoE*^−/−^ *CCN4*^+/+^ mice showed no differences in the numbers of myeloid cells in the blood, measured as the number of CD11b positive cells, or the number of monocytes, measured as CD11b + Ly6G-cells. There was also no difference in the proportion of inflammatory to non-inflammatory monocytes, measured as the number of Ly6G negative, Ly6C positive cells with either high or low Ly6C (Fig. 6 and Table 3).

## 4 Discussion

The objective of this study was to determine the role of CCN4 in atherosclerosis. We approached this by assessing the effect of elevating the plasma levels of CCN4, to observe the effects on an already established plaque, as well as deletion of CCN4 to completely remove plasma CCN4, to observe the effect on plaque development, using the fat fed *ApoE*^−/−^ mouse *in vivo* model of atherosclerosis. Our results demonstrate that in this model CCN4 has atheroprotective effects. Elevation of CCN4 resulted in decreased atherosclerotic plaque burden, while mice with no plasma CCN4 had increased plaque burden. The predominant change in plaque composition with these interventions was a change in apoptosis. In mice with elevated plasma levels of CCN4, the suppressed levels of apoptosis were associated with reduced macrophage numbers and a smaller lipid core. In mice with no CCN4 the increased apoptosis resulted in non-significant increases in macrophage number and lipid core size. There are a number of possible explanations for this, including a shorter period of high fat feeding in this study, lower overall levels of apoptosis, or compensatory upregulation of other pathways caused by the global knockout of CCN4.

We also directly demonstrated the pro-survival effect of CCN4 on macrophages in culture, with recombinant CCN4 preventing oxidised-LDL induced apoptosis, while deficiency of CCN4 in mouse macrophages resulted in a higher apoptotic rate in these cells. These observations align with our previously reported pro-survival effect of CCN4 in VSMCs, protecting them from hydrogen peroxide induced apoptosis [6]. Apoptosis is a known stimulant for macrophage recruitment [20–22], so one possibility is that the attenuation in macrophage number may be the result of lower levels of apoptosis. This leads to a reduction in lipid core size and therefore slows atherosclerotic plaque progression.

We have also previously observed that unstable human plaques contained less CCN4 compared to stable plaques [6]. The combined actions of CCN4 to reduce macrophage number and VSMC apoptosis and promote VSMC migration and proliferation could progress the plaques towards a more stable phenotype with smaller lipid cores and more robust fibrous caps.

This would reduce the likelihood of plaque rupture, which can lead to plaque growth due to incorporation of thrombus [17]. In this study CCN4 did not affect foam cell formation either when we supplemented recombinant CCN4 or in CCN4 deficient cells, therefore we conclude that the observed *in vivo* effects on plaque occlusion, lipid core size and apoptosis are most likely due to effects on apoptosis rather than any effect on foam cell formation. It is also unlikely that the observed differences in plaque macrophage numbers are dependent on differences in circulating myeloid cell populations, as no differences in the proportion of myeloid cells or inflammatory monocytes were observed.

It is important to understand the complexities of these diseases when interpreting the data and translating them into human disease. It is possible that in human atherosclerosis CCN4 may have a different effect, as human atherosclerosis may be initiated via neointimal formation rather than macrophage foam cell accumulation. However, the effects that we have observed would act to stabilise a pre-existing plaque by reducing lipid core size and therefore preserving fibrous cap thickness. We also recognise that another factor limiting the translational potential of this study is the use of only male mice; when moving forward with finding a therapeutic to stabilise atherosclerotic plaques it is important to investigate the effects in both male and female animals.

In summary, our study shows that using ApoE knockout mouse models of atherosclerotic plaque formation, CCN4 is atheroprotective. However, we know that CCN4 also has some effects that may contradict this in human atherosclerosis. To create a therapeutic, further analysis of the mechanism of action of CCN4 in atherosclerotic plaques may permit targeting of a more specific downstream effector and allow the pro-survival effect to be isolated from the pro-proliferative action of CCN4.

## Supplementary Material

Supplementary Material

## Figures and Tables

**Table 1:** Comparison of plaque characteristics and plasma lipid levels between mice infected with HD-Ad CCN4 and mice infected with control virus.

	CONTROL	CCN4 VIRUS	*p* value
Vessel IEL area (n = 15)	139,945 ± 20,393	119,515 ± 13,158	0.40
Vessel EEL area (n = 15)	199,422 ± 25,527	177,111 ± 17,614	0.47
Cell density (n = 10)	0.36 ± 0.04	0.52 ± 0.06	0.07
Proliferation (% PCNA, n = 8)	2.45 ± 0.98	1.69 ± 0.56	0.55
SMCs (% actin, n = 10)	9.72 ± 2.77	11.45 ± 2.3	0.28
Endothelial coverage (% CD31, n = 10)	38.80 ± 4.50	30.15 ± 3.37	0.18
Collagen (% picrosirius red, n = 10)	9.38 ± 4.12	7.71 ± 3.50	0.76
Total cholesterol (μg/μl)	10.5 ± 1.37	10.8 ± 1.00	0.87
LDL/vLDL (μg/μl)	2.5 ± 0.29	2.5 ± 0.24	0.91
HDL (μg/μl)	1.1 ± 0.25	0.7 ± 0.08	0.27

Area of IEL and EEL, cell density, proliferation, number of SMCs, endothelial coverage, and the amount of collagen were analysed. Plasma total cholesterol and LDL/vLDL and HDL (μg/μl) were measured. Values are shown as mean ± sem with *t*-test or Mann-Whitney test.

**Table 2:** Comparison of plaque characteristics and plasma lipid levels between CCN4-deficient mice and controls.

	*CCN4* ^+/+^	*CCN4* ^−/−^	*p* value
Vessel IEL area (n = 13)	150,321 ± 14,339	154,702 ± 13,059	0.86
Vessel EEL area (n = 13)	232,956 ± 20,952	227,817 ± 17,089	0.84
Cell density (n = 8)	0.32 ± 0.05	0.18 ± 0.04	0.06
Proliferation (% PCNA, n = 8)	9.57 ± 2.89	2.69 ± 1.02	0.05
SMCs (% actin, n = 8)	6.51 ± 1.80	8.30 ± 2.40	0.55
Endothelial coverage (% CD31, n = 10)	41.19 ± 6.23	59.77 ± 6.16	0.05
Collagen (% picrosirius red, n = 10)	9.78 ± 4.26	21.32 ± 8.53	0.23
Total cholesterol (μg/μl)	10.7 ± 1.28	10.0 ± 1.19	0.70
LDL/vLDL (μg/μl)	1.6 ± 0.33	1.5 ± 0.21	0.72
HDL (μg/μl)	1.1 ± 0.35	0.9 ± 0.14	0.59

Area of IEL and EEL, cell density, proliferation, number of SMCs and macrophages, endothelial coverage, and the amount of collagen were analysed. Plasma total cholesterol and LDL/vLDL and HDL (μg/μl) were measured. Values are shown as mean ± sem with *t*-test or Mann-Whitney test.

**Table 3:** Comparison of blood leukocytes between CCN4-deficient mice and controls.

	*CCN4*^+/+^ (n = 4)	*CCN4*^−/−^ (n = 4)	*p* value
Total cells	55163 ± 20437	22589 ± 7407	0.20
CD11b + cells (myeloid cells)	6722 ± 2806	3866 ± 1405	0.49
CD11b + Ly6G- cells (monocytes)	6043 ± 2502	3257 ± 1058	0.34
Ly6G + cells (neutrophils)	302 ± 107	245 ± 129	0.60
Ly6C high cells (inflammatory)	1700 ± 654	1378 ± 298	0.66
Ly6C low cells (non-inflammatory)	708 ± 195	744 ± 131	0.66
CD11b + as % of total cells	12.5 ± 1.6	16.4 ± 1.1	0.11
CD11b + Ly6G- as % of total cells	11.4 ± 1.6	14.3 ± 1.0	0.34
Ly6C High as % of Ly6C + cells	67.2 ± 4.2	63.5 ± 3.5	0.34
Ly6C Low as % of Ly6C + cells	32.8 ± 4.2	36.5 ± 3.5	0.34

Flow cytometry was conducted using a panel of antibodies including CD11b and Ly6C to determine proportion of leukocytes (CD11b+), monocytes (CD11b + Ly6G-) and inflammatory (high Ly6C) and non-inflammatory (low Ly6C) monocytes. Values are shown as mean ± sem with Mann-Whitney test.
