# Supplementary material for: CCN4 (WISP-1) reduces apoptosis and atherosclerotic plaque burden in an ApoE mouse model
Source: Atherosclerosis. Author manuscript; Available in PMC 2025 Feb 24. (PMC7617386; doi:10.1016/j.atherosclerosis.2024.118570)
Supplement: Supplementary Material [file EMS203080-supplement-Supplementary_Material.pptx]

## Slide 1
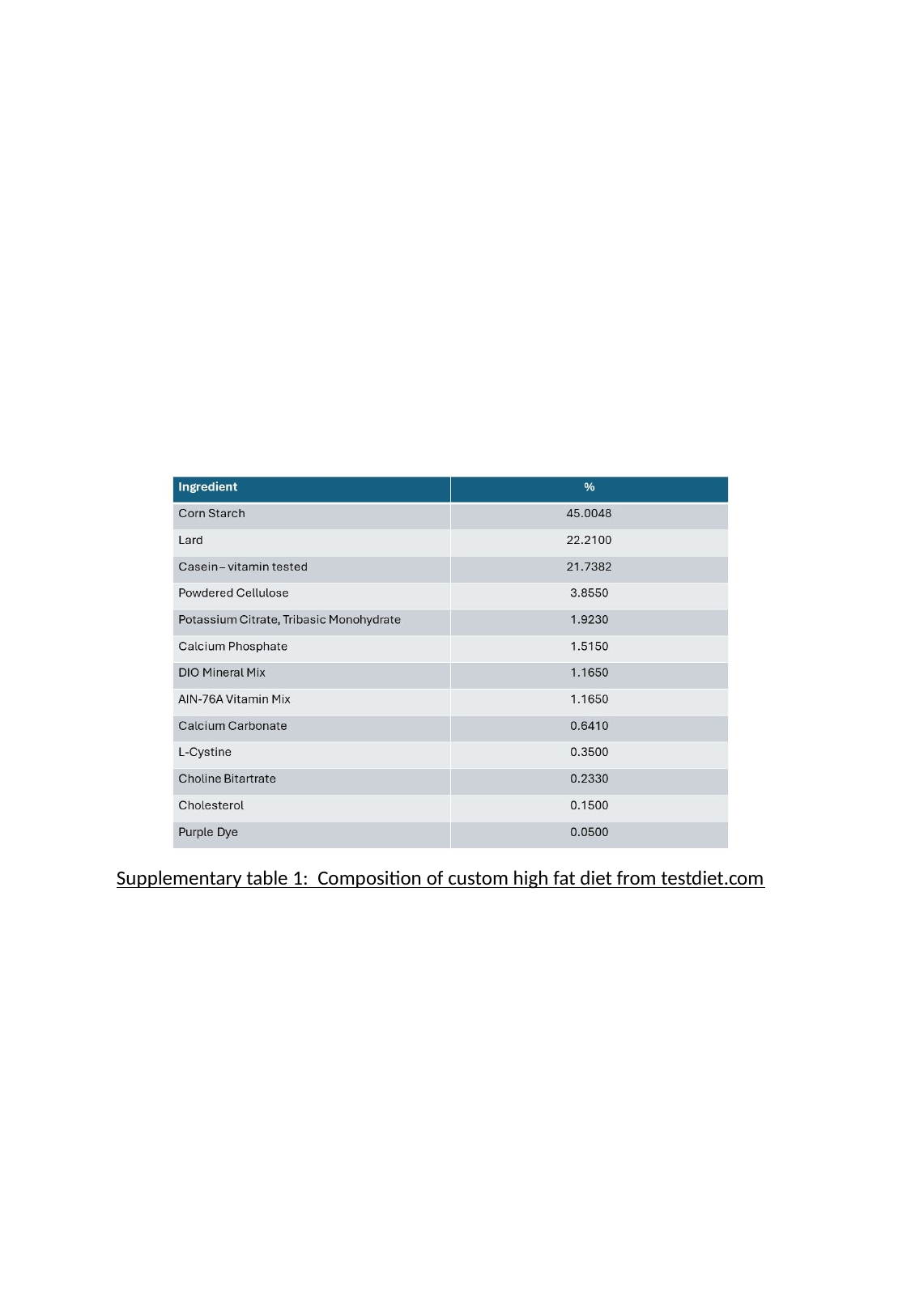

Supplementary table 1: Composition of custom high fat diet from testdiet.com

## Slide 2
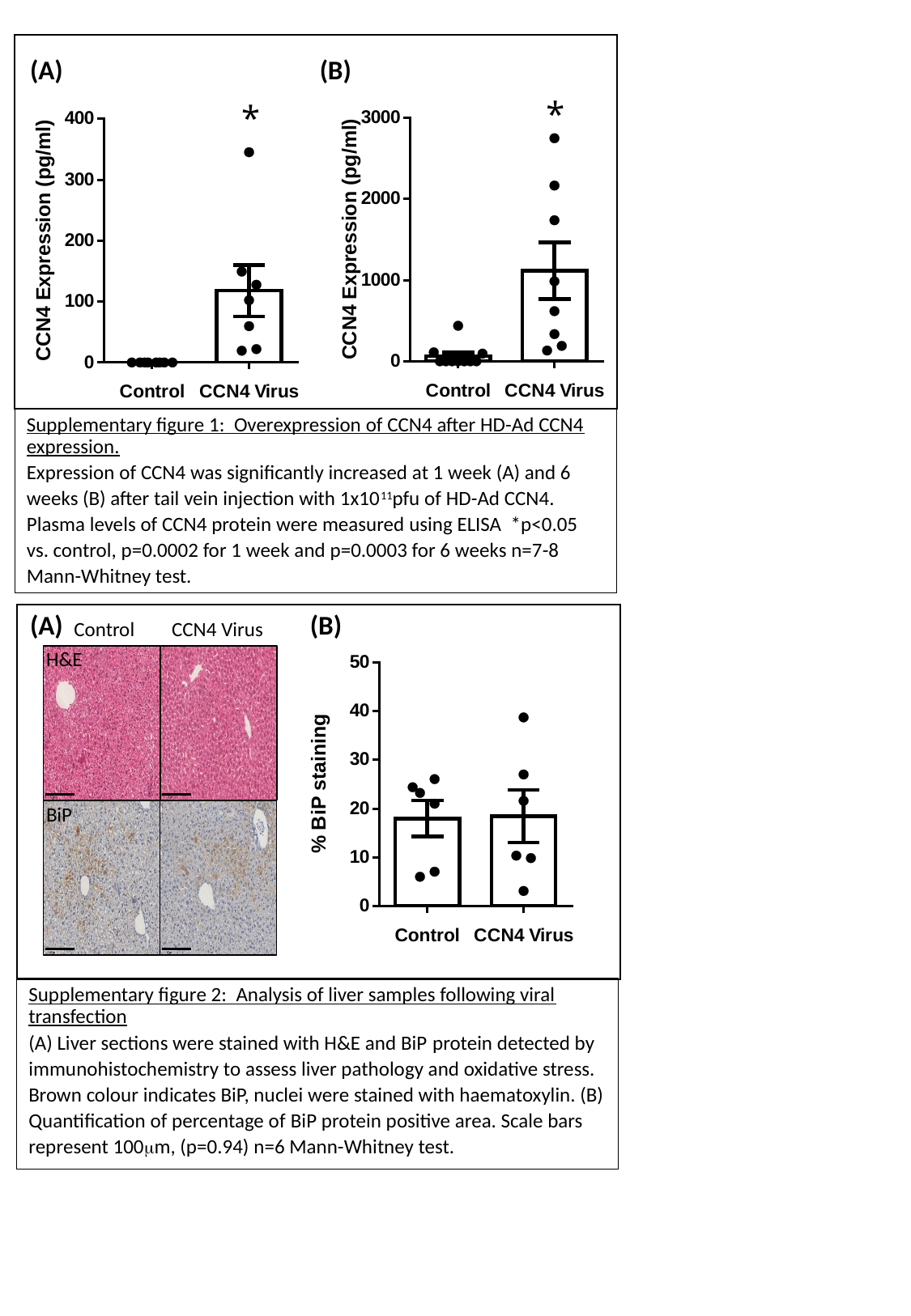

(A)
(B)
*
*
Supplementary figure 1: Overexpression of CCN4 after HD-Ad CCN4 expression.
Expression of CCN4 was significantly increased at 1 week (A) and 6 weeks (B) after tail vein injection with 1x10^11^pfu of HD-Ad CCN4. Plasma levels of CCN4 protein were measured using ELISA *p<0.05 vs. control, p=0.0002 for 1 week and p=0.0003 for 6 weeks n=7-8 Mann-Whitney test.
(A)
(B)
Control CCN4 Virus
H&E
BiP
Supplementary figure 2: Analysis of liver samples following viral transfection
(A) Liver sections were stained with H&E and BiP protein detected by immunohistochemistry to assess liver pathology and oxidative stress. Brown colour indicates BiP, nuclei were stained with haematoxylin. (B) Quantification of percentage of BiP protein positive area. Scale bars represent 100mm, (p=0.94) n=6 Mann-Whitney test.
